# An Eco-Friendly Method to Synthesize Potent Antimicrobial Tricyclic Flavonoids

**DOI:** 10.3390/antibiotics13090798

**Published:** 2024-08-24

**Authors:** Loredana-Elena Mantea, Cristina-Veronica Moldovan, Mihaela Savu, Laura Gabriela Sarbu, Marius Stefan, Mihail Lucian Birsa

**Affiliations:** 1 Department of Biology, Faculty of Biology, Alexandru Ioan Cuza University of Iasi, Bd. Carol I, No. 11, 700506 Iasi, Romania; mantealoredana9@gmail.com (L.-E.M.); mcristina.veronica@gmail.com (C.-V.M.); mihaelasavu2@gmail.com (M.S.); 2 Department of Chemistry, Alexandru Ioan Cuza University of Iasi, No. 11 Carol I Blvd., 700506 Iasi, Romania; laura.sarbu@uaic.ro

**Keywords:** antimicrobial resistance, novel antimicrobials, green chemistry, synthetic flavonoids, dithiocarbamates

## Abstract

The rapid emergence and spread of multidrug-resistant microorganisms is threatening our ability to treat common infections, with serious medical, social, and economic consequences. Despite substantial progress in the global fight against antibiotic resistance, the number of effective antibiotics is rapidly decreasing, underlying the urgent need to develop novel antimicrobials. In the present study, the green synthesis of novel iodine-substituted tricyclic flavonoids has been accomplished using an eco-friendly reagent, HPW-SiO_2_, as a cyclization agent for the precursor 3-dithiocarmamic flavanones. In vitro antimicrobial activity of the new compounds was evaluated using minimum inhibitory concentration (MIC) and minimum bactericidal/fungicidal concentrations. All tested compounds displayed potent inhibitory activity against all tested microbial strains, with the lowest MIC values of 0.12 µg/mL and 0.48 µg/mL recorded for compound **5c** against Gram-positive bacteria *Bacillus subtilis* and *Staphylococcus aureus*. Higher MIC values (7.81 µg/mL) were registered for several flavonoids against Gram-negative bacteria *Escherichia coli* and *Acinetobacter pittii*. No inhibitory activity was evidenced against *Pseudomonas aeruginosa* strain. The highest antifungal activity was displayed by flavonoid **5d** against *Candida krusei* (MIC = 3.9 µg/mL). The same compound also exhibited the most potent bactericidal and fungicidal activity against *Bacillus subtilis* (0.9 µg/mL) and *Staphylococcus aureus* (1.97 µg/mL), *Candida albicans,* and *Candida krusei* (7.81 µg/mL). Based on the reported results, we believe that the novel iodine-substituted tricyclic flavonoids have good potential to become new antimicrobial agents effective against bacterial and fungal strains, including WHO-priority pathogens.

## 1. Introduction

With an estimated number of 4.95 million deaths associated with bacterial antibiotic resistance in 2019 [1], antimicrobial resistance (AMR) is considered by the World Health Organization (WHO) to be one of the most important public health menaces. Among the main causes of AMR are the overuse and misuse of antibiotics in everyday human treatment, hospital-acquired infections associated with medical procedures, and large-scale agriculture, as well as the lack of new classes of antibiotics. Without adequate measures, it is expected that the global burden of AMR will increase considerably, with an estimated 10 million deaths per year globally by 2050 [2]. AMR is not only a medical threat but also a serious global socioeconomic problem and is expected to cause a loss of approx. 3.8% of the global gross domestic product by 2050, as predicted by the World Bank [3].

Resistant microorganisms can survive and even multiply in the presence of currently used antibiotics [4]. As a result, antimicrobial drugs become less effective, and many infections become more difficult, if not impossible, to treat, increasing the pressure on healthcare systems all around the world [5]. Moreover, the rapid emergence and spread of multidrug-resistant (MDR) microorganisms or superbugs has become a formidable challenge for modern medicine, resulting in an increased frequency of nosocomial infections [6]. The magnitude of antibiotic resistance was highlighted by many clinical studies, justifying the need for implementing antibiotic stewardship programs [7].

Despite substantial progress in the global fight against AMR, the antibiotic pipeline is drying out and therefore very few therapeutic solutions are still available against MDR strains. According to a recent WHO report, since 2017, only 13 new antibiotics have been authorized, and only two (vaborbactam and lefamulin) belong to new classes of antibiotics. Moreover, the newly approved drugs do not exhibit new mechanisms of action or cellular targets [8]. This alarming trend, together with the rise of superbugs, underscores the urgent need for innovative approaches to develop novel antimicrobials that are efficient in treating infectious diseases.

Flavonoids are a class of secondary metabolites found in fruits, vegetables, and certain beverages. Their structural diversity, characterized by a common flavone backbone with various substitutions, endows them with a broad range of biological activities, including antitumor, antioxidant, anti-inflammatory, and antimicrobial properties [9]. The antimicrobial potential of natural flavonoids has been well documented, with numerous studies highlighting their ability to disrupt microbial cell walls and membranes, interfere with nucleic acid synthesis, and inhibit essential bacterial enzymes [10]. Despite the promising antimicrobial potential of natural flavonoids, their clinical application has been limited by factors such as poor solubility, stability, and bioavailability [11]. To overcome these challenges, flavonoid derivatives have been designed and synthesized as a promising alternative to fight infectious diseases due to their enhanced antimicrobial efficacy [12].

The production of these new pharmaceuticals by conventional chemical methods exhibits a negative environmental impact through the generation of hazardous waste, the use of toxic solvents, increased energy consumption, and dependence on finite fossil resources. Green chemistry offers several advantages over conventional chemical synthesis by targeting the design of products and processes that reduce waste production and toxicity, promote the use of renewable resources, and increase energy efficiency, contributing to environmental, economic, and health benefits [13,14,15,16].

In the last decade, we have developed a new class of sulfur-containing tricyclic flavonoids with different halogen substituents at the benzopyran core. We successfully showed that these synthetic flavonoids exhibit potent antibacterial and antifungal properties, and we investigated their mechanism of action [12,17,18]. In order to minimize the environmental impact of the synthetic process, we decided to search for alternative synthetic methods. In this context, we investigated a mild and environmentally friendly approach for the synthesis of iodine-containing substrates [19]. We report here an eco-friendly method to perform the cyclization of iodine-substituted 3-dithiocarbamic flavanones. This is based on the use of a supported heteropolyacid (HPA) known to exhibit a high acidity nearing the concept of superacids and recyclability. The role of HPA as an efficient catalyst for various organic transformations is supported by literature data [20,21,22]. The newly synthesized compounds were investigated for in vitro antimicrobial activity against bacterial and fungal strains, including resistant strains identified in 2024 by the WHO as priority pathogens.

## 2. Results and Discussion

### 2.1. The Synthesis of Tricyclic Flavonoids

**Scheme 1:**
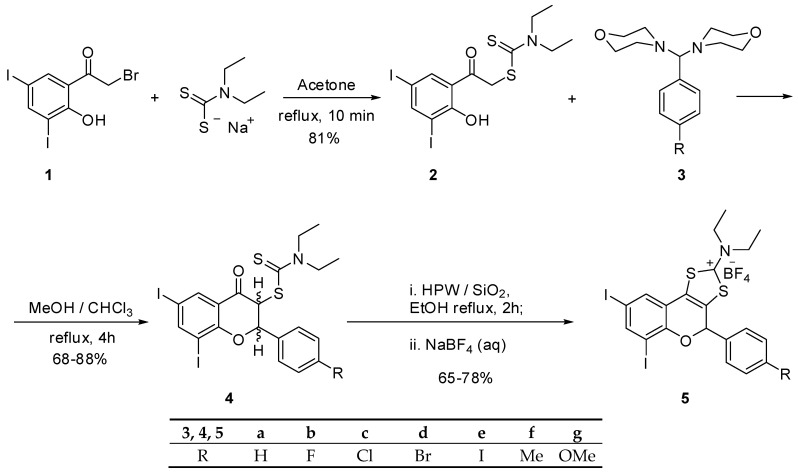
The synthesis of tricyclic flavonoids **5a**–**g**.

The synthesis of 1,3-dithiolium flavonoids **5a**–**g** has been accomplished as described in Scheme 1 by the cyclocondensation 3-dithiocarbamic flavonoids of type **4**. By reacting 2-bromo-1-(2-hydroxy-3,5-diiodophenyl)ethan-1-one (**1**) [23] with sodium *N*,*N*-diethyldithiocarbamate, in acetone, the phenacyl carbodithioate **2** has been obtained in 81% yield. The structure of the *N*,*N*-diethyldithiocarbamate **2** has been confirmed by NMR spectral data. The ^1^H NMR spectrum indicates for compound **5e** the presence of *N*,*N*-diethylamino moiety with the characteristic signal at ca. 1.3 ppm and 4 ppm. The presence of the two new methyl groups is confirmed by the ^13^C NMR spectrum at 11.4 ppm and 12.7 ppm and also of the two nitrogen-bounded methylene groups (47.5 ppm and 50.7 ppm). The thiocarbonyl carbon atom is located at 192.7 ppm.

3-Substituted dithiocarbamic flavanones **4a**–**g** have been obtained by the reaction of 1-(2-hydroxy-3,5-diiodophenyl)-1-oxaethan-2-yl *N*,*N*-diethylaminocarbodithioate (**2**) with aminals **3**, as a mixture of diastereoisomers (Scheme 1). Pale yellow precipitates were formed after cooling, which were further filtered, dried, and recrystallized from ethanol to provide 3-dithiocarbamic flavanones **4a**–**g**, in 68–82% yields. Spectral data support the formation of the benzopyran ring. There can be observed the NMR pattern of a *para*-substituted aromatic ring originating from aminal **3**. The disappearance of the characteristic signal of the methylene group from dithiocarbamate **2** (4.84 ppm) and the appearance of vicinal coupling between hydrogen atoms from the C-2 and C-3 positions of the benzopyran ring for both diastereoisomers between 5.7 and 6 ppm have been recorded.

A general method for the synthesis of 2-dialkylamino-1,3-dithiolium-2-yl cations consists of heterocyclization of the corresponding dithiocarbamates. This is accomplished under acid-catalyzed conditions. Being aware of the sensitivity of our iodine-substituted derivatives **4a**–**g** we decided to investigate an alternative method for their cyclization. 12-Tungstophosphoric acid, H_3_PW_12_O_40_·nH_2_O (HPW) is an eco-friendly catalyst employed for dehydration reactions. For this purpose, we have used HPW-SiO_2_ as a catalyst for the heterocyclocondensation of dithiocarbamates **4**. HPW supported on silica has been prepared by dissolving H_3_PW_12_O_40_·nH_2_O in 50% aqueous ethanol at room temperature. A 20% HPW-SiO_2_ was prepared with appropriate loading of the above HPW solution on the silica support. Then, the catalyst was dried and calcinated at 200 °C for 5 h. Thus, after 4 h of reflux in ethanol and the addition of sodium tetrafluoroborate following Celite 545 filtration, tricyclic flavonoids **5** have been successfully obtained.

The spectral data also confirms the structure of tricyclic flavonoids **5**. Thus, IR spectroscopy indicates the absence of the carbonyl absorption bands (1690–1710 cm^−1^) and the presence of new strong and broad absorption bands (ca. 1070–1090 cm^−1^) that belong to the tetrafluoroborate anion. The ^1^H NMR spectra of tricyclic flavonoid **5** show the disappearance of the doublet corresponding to the C-3 hydrogens. The ^13^C NMR spectra indicate the absence of the carbonyl and thiocarbonyl atoms and show a new signal at ca. 185 ppm, corresponding to the C-2 carbon atom from the 1,3-dithiol-2-ylium ring.

### 2.2. Flavonoids ***5a***–***g*** Exhibit Potent Antimicrobial Activity

All synthetic flavonoids exhibited important inhibitory activity against tested microbial strains as shown in Table 1, with one exception—*Pseudomonas aeruginosa* PAO1 for which the antibacterial properties (MIC = 125 µg/mL) were similar to DMSO used as negative control. The lowest MIC values were recorded against *Bacillus subtilis* (0.12 µg/mL) and *Staphylococcus aureus* (0.48 µg/mL), with compound **5c** being most active. The Gram-negative bacteria were less sensitive to synthetic compounds compared to the Gram-positive strains, with MICs ranging from 7.81 to 62.50 µg/mL. The tricyclic flavonoids **5a** and **5d** showed the highest antibacterial activity against *Escherichia coli* and *Acinetobacter pittii* strains. A promising inhibitory activity was also evidenced against the tested fungal strains at concentrations as low as 3.90 µg/mL recorded for **5d** against *Candida krusei* Prx (Table 1).

Important bactericidal activity was registered for flavonoid **5e** against *B. subtilis* strain, with MBC values as low as 0.48 µg/mL. The MBCs ranged from 15.62 µg/mL to 62.50 µg/mL for all synthetic flavonoids against Gram-negative bacteria, with *Pseudomonas aeruginosa* PAO1 as the less susceptible microorganism (MBC of 125 and 250 µg/mL). Significant fungicidal activity was recorded for all compounds against both tested *Candida* strains, the lowest MFC values (7.81 µg/mL) being recorded for **5c**, **5d** and **5f**—Table 2.

Our results showed that all strains were susceptible in vitro to the newly synthesized compounds, except for *Pseudomonas aeruginosa* PAO1. The important antimicrobial activity of the synthetic flavonoids **5a**–**g** was also revealed by the comparison with commercial antibiotics and antifungals used as controls. Thus, all tested compounds exhibited more potent inhibitory activity against the *Staphylococcus aureus* strain compared with ampicillin (up to 4-fold higher) and chloramphenicol (up to 16-fold higher). Compared to chloramphenicol, all compounds displayed a higher antibacterial effect (between 2 and 4-fold higher) against a clinical isolate of *Enterococcus faecium* resistant to several antibiotics. Compounds **5a** and **5d** showed similar activity to kanamycin against *E. coli* strain, while all flavonoids exhibited more pronounced inhibitory activity compared to ampicillin, except **5e**, for which the recorded MIC value was equal to the control antibiotic. A significantly higher antibacterial activity was evidenced for flavonoids **5a**–**g** against *Acinetobacter pittii* compared to ampicillin; however, all synthetic compounds showed lower activity compared with ciprofloxacin. Regarding the bactericidal activity, lower MBC values were recorded for all compounds against *S. aureus*, *B. subtilis*, *E. faecium*, *E. coli,* and *A. pittii* strains.

Compared to Panduratin A, one of the most potent natural flavonoids described before (MICs between 0.5 and 1 µg/mL), compounds **5a**–**g** exhibited a similar inhibitory activity against *S. aureus* [24]. In relation to other synthetic flavonoids, our compounds were up to 520-fold more active against *S. aureus* and up to 46-fold more active against *E. coli* strains compared to previously reported chalcones, flavanones, and flavanones with chlorine, bromine, and methoxy groups as substituents [25]. Also, they displayed stronger anti-*S. aureus* potential (up to 33-fold) compared to different substituted chalcones containing nitro, amino, hydroxyl, methoxy, and chloro groups with MICs ranging from 1 to 16.76 µg/mL. However, a lower inhibitory activity compared to the same chalcone derivatives was recorded against *E. coli* (MICs from 1 to 4.85 µg/mL) and *P. aeruginosa* (MICs from 1.16 to 2.43 µg/mL) [26]. Compared to 2R-sophoflavonoid A (a new prenylated flavonoid), fluorinated chalcone-1,2,3-triazole hybrids (MIC = 0.5–1 µg/mL), β-chlorovinyl chalcones, or different chlorinated chalcones (MIC = 2 µg/mL), **5a**–**g** showed also a superior ability to suppress the growth of *S. aureus* [27,28,29,30].

All **5a**–**g** flavonoids expressed significantly higher antifungal activity against *C. albicans* and better activity against *C. krusei* strains compared to fluconazole, with the recorded MIC values being up to 64-fold smaller compared to the tested antifungal. Compared to natural flavonoids such as common flavones and isoflavone (MIC = 15.62 and 7.81 μg/mL, respectively), myricetin (MIC up to 64 μg/mL); quercetin (MIC up to 441 mg/mL), all **5a**–**g** compounds showed similar or higher activity against *Candida* spp. [31,32]. Also, the tested synthetic flavonoids inhibited the growth of *Candida* spp. at similar or lower concentrations compared to pyrazine analogs of chalcones (MIC = 7.81 μg/mL), 1,4-disubstituted-1,2,3-triazole derivatives of chalcones (MIC = 6.5–12.5 μg/mL), 2′4′-dihydroxychalcone (MIC = 15.6 μg/mL) or oxathiolone-fused chalcone derivative (MIC values up to 16 μg/mL) [33,34,35,36].

We need to emphasize that an MBC(MFC)/MIC ratio between 1 and 4 was calculated for all compounds, evidencing bactericidal/fungicidal activity against most of the strains. An exception occurred for compounds **5b,c** and **5e** against *S. aureus*, for which bacteriostatic activity was evidenced.

The excellent antimicrobial activity may be attributed to the formation of a supplementary 1,3-dithiolium ring on the **5a**–**g** flavonoids scaffold. It is well known that the C-2 position of the 1,3-dithiolilic ring is prone to nucleophilic substitution. The potent effect is most probably related to the interactions between the electrophilic C-2 atom of this ring and the nucleophilic moieties of the cellular wall and membrane constituents such as peptides or substituted polysaccharides, as we previously showed [17]. We may presume that the positively charged 1,3-dithiolium flavonoids target the negatively charged molecules from the cell wall or membrane structure, such as phosphatidylethanolamine. The lower susceptibility of the Gram-negative bacteria to **5a**–**g** flavonoids may be explained by the existence of an additional outer membrane in the cell wall structure, which acts as a barrier to protect the cell from the harmful effects of antimicrobials [37].

Our previous studies [12,17] showed that the main mechanism of action of sulfur-containing tricyclic flavonoids with halogen substituents at the benzopyran core is related to membrane integrity impairment for both bacterial and fungal cells. However, further investigations are necessary to confirm this mechanism of action for **5a**–**g** compounds.

We must highlight that inhibition of growth and bactericidal/fungicidal effects were recorded against resistant microbial strains. Some of them are included on the 2024 WHO priority pathogens list: *E. coli* or *Acinetobacter* spp. (considered as critical) or *S. aureus* and *E. faecium* (members of the high group); others are emerging as important human pathogens, such as *C. albicans* and *C. krusei*. All these findings suggest that flavonoids **5a**–**g** are compounds with valuable antimicrobial activity, comparable to both flavonoids and antibiotics/antifungals.

## 3. Materials and Methods

### 3.1. Chemistry

Melting points were obtained on a *KSPI* melting-point meter (A. KRÜSS Optronic, Hamburg, Germany) and are uncorrected. IR spectra were recorded on a Bruker Tensor 27 instrument (Bruker Optik GmbH, Ettlingen, Germany). NMR spectra were recorded on a Bruker 500 MHz spectrometer (Bruker BioSpin, Rheinstetten, Germany). Chemical shifts are reported in ppm downfield from TMS. Mass spectra were recorded on a Thermo Scientific ISQ LT instrument (Thermo Fisher Scientific Inc., Waltham, MA, USA). All reagents were commercially available and used without further purification.

#### 3.1.1. General Procedure for 6,8-diiodo-2-(4-iodophenyl)-4-oxochroman-3-yl *N*,*N*-diethyldithiocarbamate (**4e**)

To a solution of 1-(3,5-diiodo-2-hydroxyphenyl)-1-oxoethan-2-yl *N*,*N*-diethyldithiocarbamate (**2**) (0.268 g, 0.5 mmol) in a mixture of CHCl_3_/MeOH (12 mL, 1:1 *v*/*v*) aminal **3e** (0.195 g, 0.5 mmol) was added and the reaction mixture was heated under reflux for 4 h. After cooling, the solid material was filtered off and purified by recrystallization from ethanol to give **4e** (0.3 g, 82%) as colorless crystals. M.p. 186–187 °C. IR (ATR, cm^−1^) 2741, 1680, 1415, 1250, 1188, 975, 808, 501, 481. ^1^H NMR (CDCl_3_, selected data for the major isomer) δ 8.28 (d, *J* = 2.0 Hz, 1H), 8.16 (d, *J* = 2 Hz, 1H), 7.71 (d, *J* = 8.4 Hz, 2H), 7.26 (d, *J* = 8.4 Hz, 2H), 6.26 (d, *J* = 6.1 Hz, 1H), 5.96 (d, *J* = 6.1 Hz, 1H), 3.91 (m, 2H), 3.69 (m, 2H), 1.26 (m, 6H). ^13^C NMR (CDCl_3_, selected data for the major isomer) δ 190.8, 185.9, 158.6, 152.7, 137.6, 136.5, 135.5, 129.3, 128.8, 122.8, 95.0, 87.4, 85.1, 82.6, 81.0, 58.1, 50.7, 47.4, 12.6, 11.4. MS (EI) *m/z*: 748.8 (M^+^, 14%) for C_20_H_18_I_3_NO_2_S_2_.

#### 3.1.2. General Procedure for 2-*N*,*N*-Diethylamino-6,8-diiodo-4-(4-iodophenyl)-4*H*-1,3-dithiol[4,5-*c*]chromen-2-ylium Tetrafluoroborate (**5e**)

Flavanone **4e** (0.188 g, 0.25 mmol) and HPW-SiO_2_ have been suspended in ethanol and heated at reflux for 5 h. The reaction mixture was filtered hot through Celite 545 and after cooling to room temperature aqueous NaBF_4_ was added under vigorous stirring. The resulting precipitate was then filtered, washed thoroughly with water and recrystallized from ethanol, yielding the target tetrafluoroborate **5e** in the form of colorless crystals (0.165 g, 81%). M.p. 247–248 °C. IR (ATR, cm^−1^) 1570, 1430, 1219, 1047, 732, 499, 457. ^1^H NMR (DMSO-*d*6) δ 8.01 (d, *J* = 1.8 Hz, 1H), 7.76 (d, *J* = 1.8 Hz, 1H), 7.39 (d, *J* = 8.2 Hz, 2H), 7.24 (d, *J* = 8.2 Hz, 2H), 6.84 (s, 1H), 3.87 (m, 4H), 1.41 (t, *J* = 7.2 Hz, 3H), 1.31 (t, *J* = 7.2 Hz, 3H). ^13^C NMR (DMSO-*d*6) δ 185.1, 150.3, 147.9, 136.8, 133.4, 132.4, 130.2, 128.8, 127.2, 124.1, 95.4, 88.6, 87.7, 75.4, 54.4, 54.5, 10.6, 10.4. MS (EI) *m/z*: 731.8 (M^+^-BF_4,_ 5%) for C_20_H_17_I_3_NOS_2_]^+^.

The spectral data of tricyclic flavonoids **5a**–**d** and **5f,g** are in accordance with the previous reported data [19].

### 3.2. Microbial Strains and Culture Conditions

The following strains were used for antimicrobial assay: *Staphylococcus aureus* ATCC 25923, *Bacillus subtilis* ATCC 6633, *Escherichia coli* ATCC 25922, *Pseudomonas aeruginosa* PAO1 and *Candida albicans* ATCC 10231 (obtained from the culture collection of the Microbiology Laboratory, Alexandru Ioan Cuza University of Iasi; *Acinetobacter pittii* Cl2 (resistant to ampicillin and chloramphenicol) was isolated from a local wastewater treatment plant and identified using MALDI-TOF/MS spectrometry; *Enterococcus faecium* medbio2-2012 (resistant to ampicillin, ceftazidime, ciprofloxacin, gentamicin, levofloxacin, and penicillin) and *Candida krusei* Prx (resistant to fluconazole) strains were kindly provided by med. biol. PhD Simona Matiut from Praxis Clinical Laboratory, Iasi, Romania.

All bacteria were cultured aerobically at 37 °C and 190 rpm using Mueller-Hinton agar (MHA, Accumix, Geel, Belgium) and Mueller Hinton broth (MHB, Roth, Karlsruhe, Germany); fungal strains were cultured at 37 °C and 130 rpm on Sabouraud dextrose agar (SDA, Roth, Karlsruhe, Germany) and Sabouraud dextrose broth (SDB, Roth, Karlsruhe, Germany).

Microbial strains were stored in 15% glycerol stocks at −80 °C. Before testing, the strains were cultured overnight on MHA or SDA. Afterwards, 10 mL of MHB and SDB were inoculated with one representative colony, incubated overnight and used as inoculum for each assay.

### 3.3. Antibacterial Susceptibility Testing: Determination of the Minimum Inhibitory Concentration and the Minimum Bactericidal/Fungicidal Concentration

The minimum inhibitory concentration (MIC) was determined by the broth microdilution method, as we previously described [17]. Briefly, each flavonoid was serially diluted in MHB or SDB using 96-well plates and DMSO (Roth, Karlsruhe, Germany) as solvent. The tested concentrations ranged from 0.12 to 250 µg/mL. The inoculum cell density was adjusted to approximately 2 × 10^6^ CFU/mL (CFU = colony-forming units) for bacterial suspensions and 1 × 10^3^ CFU/mL for fungal suspensions. DMSO at concentrations ranging from 0.012 to 24.87% (*v*/*v*) served as a negative control. The growth control was represented by inoculated MHB or SDB medium. Ampicillin, chloramphenicol, kanamycin, ciprofloxacin and fluconazole were used as reference antimicrobials. MIC was considered to be the lowest concentration of the tested compound with no visible microbial growth after incubation at 37 °C for 24 h. A volume of 5 µL taken from each well with no visible growth was inoculated on MHA/SDA plates to assess the minimum bactericidal concentration (MBC) and minimum fungicidal concentration (MFC), respectively. MBC or MFC were considered as the lowest concentrations with no colony growth after plating onto MHA or SDA.

Each experiment was repeated at least three times.

## 4. Conclusions

An improved and eco-friendly method for the synthesis of sensitive iodine-containing tricyclic flavonoids has been developed using a supported heteropolyacid as a catalyst. The synthesized compounds exhibited important antimicrobial activity against bacterial and fungal pathogens at low concentrations. Further studies are necessary to explore their potential as efficient antimicrobials to be used in the fight against AMR.

## Figures and Tables

**Table 1:** Minimum inhibitory concentration of flavonoids **5a**–**g** against tested microbial strains (µg/mL).

Microbial Strains	5a	5b	5c	5d	5e	5f	5g	DMSO (%)	Control
*Staphylococcus aureus* ATCC 25923	0.97	0.97	0.48	0.97	0.48	0.97	0.97	24.87	1.95 ^a^/7.81 ^chl^
*Bacillus subtilis ATCC 6633*	0.24	0.12	0.12	0.48	0.24	0.9	0.48	24.87	0.12 ^a^
*Enterococcus faecium* medbio2-2012	7.81	3.9	3.9	3.9	3.9	3.9	3.9	12.43	15.62 ^chl^
*Escherichia coli* ATCC 25922	7.81	15.62	31.25	7.81	62.50	15.62	31.25	12.43	62.50 ^a^/7.81 ^k^
*Pseudomonas aeruginosa* PAO1	125	125	125	125	125	125	125	12.43	>250 ^a^
*Acinetobacter pittii* Cl2	7.81	7.81	7.81	62.50	62.50	15.62	31.25	6.21	>250 ^a^/0.37 ^cip^
*Candida albicans* ATCC 10231	15.62	7.81	7.81	7.81	7.81	15.62	15.62	6.21	>500 ^f^
*Candida krusei* Prx	7.81	7.81	7.81	3.90	15.62	7.81	7.81	6.21	62.5 ^f^

^a^—ampicillin; ^chl^—chloramphenicol; ^cip^—ciprofloxacin; ^k^—kanamycin; ^f^—fluconazole. The values are means for at least three replicates.

**Table 2:** Minimum bactericidal/fungicidal concentration of flavonoids **5a**–**g** against tested microbial strains (µg/mL).

Microbial Strains	5a	5b	5c	5d	5e	5f	5g	Control
*Staphylococcus aureus* ATCC 25923	3.90	7.81	3.90	1.97	3.90	3.90	3.90	7.8 ^a^
*Bacillus subtilis ATCC 6633*	0.9	0.9	1.9	0.9	0.48	3.9	1.95	3.9 ^a^
*Enterococcus faecium* medbio2-2012	62.5	15.62	31.25	62.5	15.62	62.5	31.25	>250 ^chl^
*Escherichia coli* ATCC 25922	31.25	31.25	62.50	31.25	125	62.50	31.25	125 ^a^
*Pseudomonas aeruginosa* PAO1	250	250	250	125	125	125	125	>250 ^a^
*Acinetobacter pittii* Cl2	31.25	15.62	31.25	125	125	62.50	31.25	>250 ^a^
*Candida albicans* ATCC 10231	15.62	15.62	7.81	7.81	15.62	15.62	15.62	>500 ^f^
*Candida krusei* Prx	15.62	15.62	7.81	7.81	31.25	7.81	15.62	62.5 ^f^

^a^—ampicillin; ^chl^—chloramphenicol; ^f^—fluconazole. The values are means for at least three replicates.

## Data Availability

Data are contained within the article.
